# Tribological Performance of Green Lubricant Enhanced by Sulfidation IF-MoS_2_

**DOI:** 10.3390/ma9100856

**Published:** 2016-10-21

**Authors:** Shih-Chen Shi

**Affiliations:** Department of Mechanical Engineering, National Cheng Kung University (NCKU), No. 1 University Road, Tainan 70101, Taiwan; scshi@mail.ncku.edu.tw; Tel.: +886-6-275-7575 (ext. 62176); Fax: +886-6-235-2973

**Keywords:** tribology, green lubricant, MoS_2_, HPMC, sustainable manufacturing

## Abstract

Biopolymers reinforced with nanoparticle (NP) additives are widely used in tribological applications. In this study, the effect of NP additives on the tribological properties of a green lubricant hydroxypropyl methylcellulose (HPMC) composite was investigated. The IF-MoS_2_ NPs were prepared using the newly developed gas phase sulfidation method to form a multilayered, polyhedral structure. The number of layers and crystallinity of IF-MoS_2_ increased with sulfidation time and temperature. The dispersity of NPs in the HPMC was investigated using Raman and EDS mapping and showed great uniformity. The use of NPs with HPMC enhanced the tribological performance of the composites as expected. The analysis of the worn surface shows that the friction behavior of the HPMC composite with added NPs is very sensitive to the NP structure. The wear mechanisms vary with NP structure and depend on their lubricating behaviors.

## 1. Introduction

A few years ago, the concept of sustainable manufacturing was proposed for use in the manufacturing field due to environment issues. It is important to select and use suitable lubricants in the manufacturing process in order to maintain product quality, reduce wear, and save energy. To meet the requirements of environmentally sustainable development of the manufacturing process, researchers have focused their attention on sustainable manufacturing [1], green tribology [2,3], and green lubricants technology such as ionic liquid bio-lubricants [4,5,6] and bio-based polymers [7]. Natural oils have been known to have good lubricating properties and to be effective in reducing friction and avoiding wear since the 19th century. Recently, the investigation of vegetable oil [8,9], rice bran oil [10], rapeseed oil [11], and coconut oil-based lubricants [12] have gained much attention.

In addition to the liquid lubricants, dry coatings such as nitride-based hard coatings [13] and diamond-like carbon coatings [14] have been widely investigated and discussed as wear resistance lubricants. Researchers have also used certain metals [15,16], oxides [17,18], and sulfides [19], as well as diamond [20,21] as additives to enhance the lubricating properties of the original materials. Usually, adding additives of appropriate composition and proportion can effectively improve the mechanical properties of composite materials, enhancing their tribological performance [22,23,24]. There are several important factors that affect the tribological properties of composite materials, such as material, mechanical properties, morphology, and qualities of the additive particles, as well as surface roughness and size of the counter material [25,26,27,28]. Solid lubricant microparticles, glass fiber, and carbon fiber are common additives to polymer composites. These additives can affect the formation of transfer films/tribofilms, change the shape of wear debris, or increase the load capacity, thereby improving wear resistance [29,30,31]. Early research on polymer additive nanoparticles was not ideal [32]. However, this situation changed as Burris and Sawyer developed ultra-low wear PTFE nanocomposites, which can reduce wear by up to 1000 times [33]. Although disputes still exist regarding how additives improve the tribological properties of polymer composites, many studies have shown that additives affect the formation and development of the transfer layer, either by adjusting the size of wear debris by promoting the decomposition of polymer, or through the decomposition of the filler itself to increase adhesion of the transfer layer to the counter surface [34,35,36,37,38].

The inorganic fullerene (IF)-MoS_2_, which can be prepared through various processes [39,40,41], is biocompatible [42,43], and its lubricating properties have been proved to be highly effective [39,44,45]. Therefore, it can potentially be applied in sustainable manufacturing. However, the above additive nanoparticles are difficult to be applied because of their inhomogeneity of concentration and dispersion. The biocompatible HPMC, which can be applied in various applications such as anti-wear and anticorrosion [46], can effectively solve the problems caused by the inhomogeneity of concentration and dispersion of NPs [47]. Furthermore, the added hexagonal (2H)-MoS_2_ can lead to stable and high-level lubricating properties [48].

The tribology performance of different MoS_2_ (2H-MoS_2_, IF-MoS_2_) and MoO_3_ particles as additives in wet lubricants have been well studied and reported [45,49,50]. Researchers compared their tribology performance, as dry coating additives were inferior [51,52]. Thus, this study conducted further investigations into the influence of different structure of MoS_2_ particles on the lubricating properties of composite thin films. The study used dry, lubricating thin films produced from eco-friendly and environmentally friendly biological polymer material [53] to investigate the influence of 2H-MoS_2_ microparticles (MPs), IF-MoS_2_, and MoO_3_ nanoparticles (NPs) additives on its lubricating properties. We intended to improve the lubricating properties of HPMC by adding particles and further disclose the lubricating mechanism of composite materials with the different additives. The experiment aimed to understand how the size, shape, and structure of the additives affected the tribological performance of HPMC-based composite thin films.

## 2. Materials and Methods

IF-MoS_2_ was produced through the following steps: MoO_3_ NPs (400–1000 nm, 99.9%, US Research Nanomaterials Inc., Houston, TX, USA) was placed in an aluminum oxide boat. Then, it was moved into a quartz tube and mechanically pumped to 10^−3^ torr for 1 h. Then, it was filled with nitrogen gas, and the pressure was increased back to 1 atm for 10 min. Thereafter, 10% H_2_S was added at 20 standard cubic centimeters per minute (sccm) and Argon at 180 sccm, and the mixture was heated to the reaction temperature for 30–120 min for sulfidation. 2H-MoS_2_ (1–2 μm, 99%, Sigma Aldrich Chemistry, Saint Louis, MO, USA) was bought from the nano-material manufacturing company.

The thin film was produced through the following steps: 5 g of HPMC (specifications as Table 1, Pharmacoat 606, Shin-Etsu, Tokyo, Japan) were placed in 30 mL of water and 130 mL of an alcohol solution and heated to 60 °C. Then, 1.35 g NPs were added to the solution, and the mixture was vibrated via ultrasound for 20 min. Then, the solution of composite material was injected onto a silicon substrate via micropipette and held for 1 h at a temperature of 25 ± 2 °C at 60 RH ± 5% to form the thin film.

The analysis of the surface shape and element was conducted by a launched electron scanning microscope (SEM, JEOL, JSM-6700F, Peabody, MA, USA) equipped with EDS. The material structure was analyzed with a high-resolution transmission electron microscope (HRTEM, JEOL, JEM-4000EX, Peabody, MA, USA). The Raman spectrum analysis was conducted with a micro-Raman spectrometer (Renishaw, New Mills, UK). The homogeneity of the thin film was measured via the Raman spectrum technique, at which point nine points on the test piece for measurement were evenly selected, and the locations and magnitudes of the characteristic peaks in the Raman signal spectrum were compared.

The tribotest was performed via ball-on-disk in ambient air and temperature with a sliding speed of 0.01 m/s. The friction and wear tests were conducted at a rotating radius of 2 mm and a loading of 2 N. The steel balls (radius 2.5 mm, Ra = 200 nm) were made of DIN 17350. The tested particle-added HPMC composites were coated on the silicon substrate and mounted into the bottom disk of the tribometer. Friction force was measured by a load cell connected to the rotating disk. The friction coefficient was recorded for further tribological properties analysis.

A 3D scanner (Keyence, VK9710, Osaka, Japan) was used for the surface roughness and wear volume measurements.

## 3. Results and Discussion

**Figure 1:**
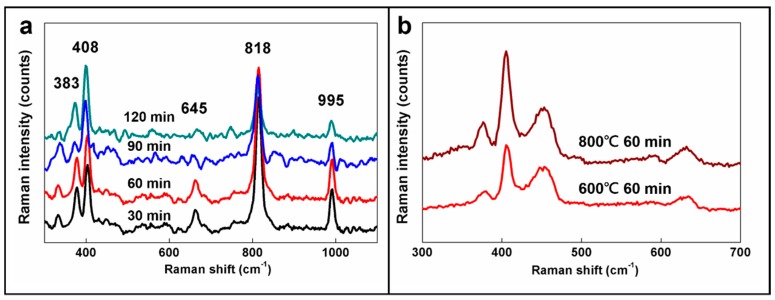
Raman analysis for IF-MoS_2_ nanoparticles (NPs) (**a**) for different sulfidation times at 600 °C and (**b**) at different sulfidation temperatures for 60 min.

Raman analysis of the NPs at the sulfidation temperature of 600 °C is shown in Figure 1a. The sulfidation of MoS_2_ could be evaluated by its five characteristic peaks at 383 cm^−1^(E^1^_2g_), 408 cm^−1^(A_1g_), 466 cm^−1^(2LA(M)), 645 cm^−1^(A_1g_ + LA(M)), and 818 cm^−1^(2A_1g_). The peak at 995 cm^−1^ is the characteristic peak of MoO_3_ [54,55]. By analyzing the highest peak at 408 cm^−1^ as an example, it was found that the peak magnitude is higher for longer sulfidation times. On the other hand, the peak magnitude at 995 cm^−1^ decreases as sulfidation time increases. This demonstrates that, at 600 °C, MoO_3_ is gradually sulfidated to MoS_2_, and the sulfidation is enhanced as process time increases. This also proves that MoO_3_ is sulfidated to MoS_2_ from the outer to the inner layer and that MoO_3_ exists in the core even after the outer layer has been sulfidated to MoS_2_. Therefore, the compatible material IF-MoS_2_/MoO_3_ can be produced using this process, which was corroborated by later TEM analysis. The influence of sulfidation temperature on IF-MoS_2_ was further analyzed and is shown in Figure 1b. The fact that the characteristic peaks of MoS_2_ are enhanced as sulfidation temperature increases demonstrates that sulfidation is faster at a higher temperature; therefore, the produced IF-MoS_2_ has a higher number of layers.

**Figure 2:**
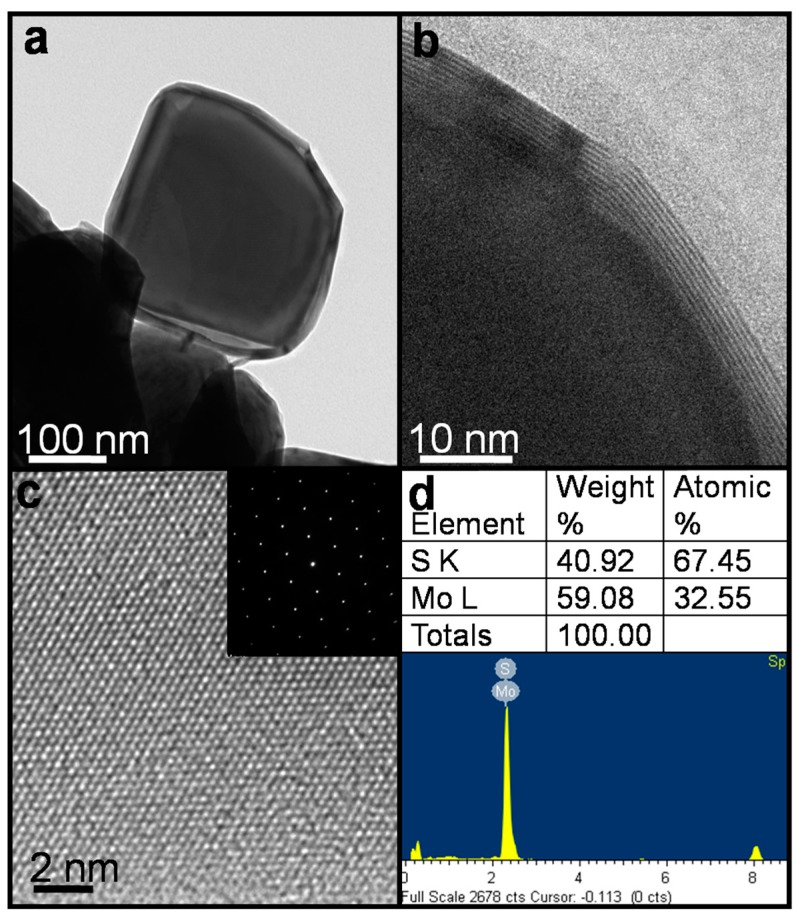
TEM analysis of IF-MoS_2_ NP: (**a**) low-resolution image; (**b**) high-resolution image; (**c**) high-resolution TEM image of MoS_2_ lamellar structure with clear lattice images. The inset shows diffraction pattern at a selected area; (**d**) Energy dispersive spectrometry (EDS) analysis of MoS_2_ lamellar structure.

The profile of the nanoparticle is shown in the low-resolution TEM image in Figure 2a. The eight-layered structure of the spherical substrate is displayed in the high-resolution TEM image in Figure 2b. The layer structure was analyzed further and is shown in Figure 2c; the diffraction pattern and crystal lattice in the selected area show the structure of MoS_2_. The EDS analysis shown in Figure 2d also confirms the structure of MoS_2_, which lies on the outside of the MoO_3_ substrate. The results of the TEM analysis and Raman spectrum analysis are in agreement. The properties of the IF-MoS_2_ produced by the gas phase sulfidation coincides with that obtained by Tenne et al. [56,57]. Therefore, its excellent lubricating properties can be predicted.

**Figure 3:**
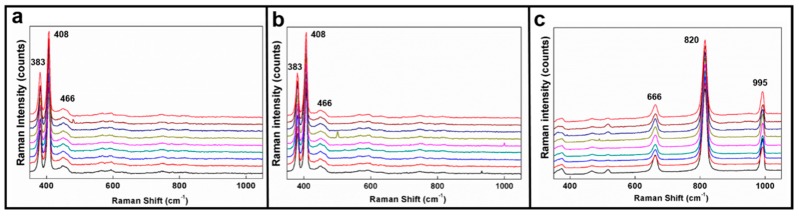
Nine-point Raman analysis of homogeneity for (**a**) IF-MoS_2_ NP-; (**b**) 2H-MoS_2_ NP-; and (**c**) MoO_3_ NP-added HPMC.

With an excellent dispersity of additives in HPMC, this study experimentally solved the problems caused by the inhomogeneity of the concentration and agglomeration of NPs in thin films. The dispersity of particles was tested by the nine-point Raman spectrum method [43], and the locations and magnitudes of three characteristic peaks are labeled at 383 cm^−1^(E^1^_2g_), 408 cm^−1^(A_1g_), and 466 cm^−1^(2LA(M)) in Figure 3a,b [54]. The results demonstrate that the locations of characteristic peaks overlap, and the difference among the magnitudes is less than 5%. This substantiates the excellent dispersity and homogeneity of additives in HPMC. The Raman signal spectra of the HPMC thin film with added MoO_3_ NPs is shown in Figure 3c. The excellent dispersity and homogeneity of NPs was confirmed by comparing the locations and magnitudes of characteristic peaks of MoO_3_ at 666 cm^−1^(B_2g′_B_3g_), 820 cm^−1^(A_g′_B_1g_), 995 cm^−1^(A_g′_B_1g_) [54], etc. Overall, the results displayed in Figure 3 indicate that the coating achieved excellent structure and crystallinity after NPs were added.

**Figure 4:**
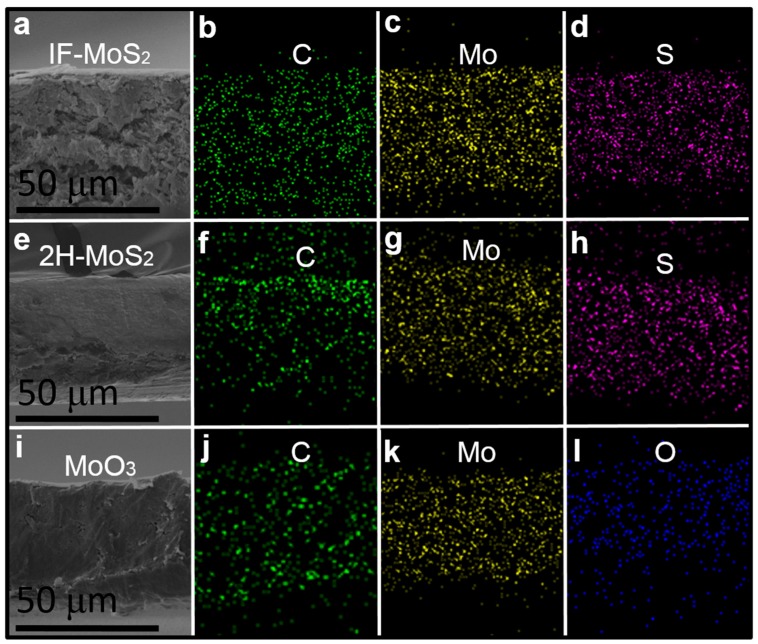
(**a**) SEM cross-sectional image with IF-MoS_2_ NP additives; EDS mapping results of the (**b**) C signal, (**c**) Mo signal, and (**d**) S signal; (**e**) SEM cross-sectional image with 2H-MoS_2_ NP additives; EDS mapping results of the (**f**) C signal, (**g**) Mo signal, and (**h**) S signal; (**i**) SEM cross-sectional images with MoO_3_ NP additives; EDS mapping results of the (**j**) C signal, (**k**) Mo signal, and (**l**) O signal.

Figure 4a,e,i show the cross-sectional SEM images with IF-MoS_2_, 2H-MoS_2_, and MoO_3_ additives. The results demonstrate their high thickness-control capability. Furthermore, energy dispersive spectrometry (EDS) mapping was performed in the present study to observe the element distribution of the coating, which is displayed in Figure 4. Figure 4b–d respectively present results of the EDS element mapping of the C, Mo, and S signals on the IF-MoS_2_-added HPMC-coated Si surface. Here, C is from HPMC, Mo and S are from MoS_2_, and O is from MoO_3_. Figure 4f–h respectively present results of the EDS element mapping of the C, Mo, and S signals on the 2H-MoS_2_-added HPMC-coated Si surface. Figure 4j–l respectively present results of the EDS element mapping of the C, Mo, and O signals on the MoO_3_-added HPMC-coated Si surface. This figure illustrates that C, Mo, S, and O are completely and uniformly distributed across the coating, which demonstrates the high dispersity of additives in thin films. Therefore, it substantiates the prediction that HPMC can be used to solve the problems caused by the inhomogeneity of the concentration and dispersion of additives.

MoS_2_ particles are well-known as a solid lubricant, the appropriate addition of which can provide load capacity and enhance the MoS_2_ lubricating layer in the contact area [23,24]. Because the addition of MoS_2_ can significantly improve strength and mechanical properties, friction behavior of the composites is primarily characterized by the effectiveness of MoS_2_ [48]. To investigate wear behavior in this experiment, SEM images were used to study how particle structure affected surface wear during the tribotest.

**Figure 5:**
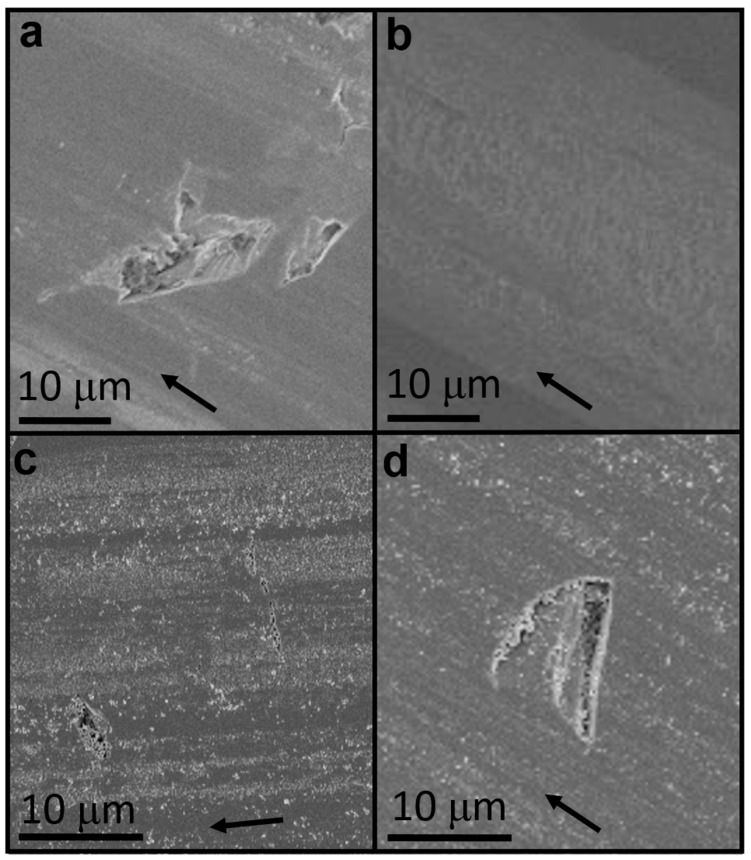
SEM image of worn surface. (**a**) Pure HPMC and (**b**) IF-MoS_2_-; (**c**) 2H-MoS_2_-; and (**d**) MoO_3_-added HPMC. The arrows show the sliding direction.

Figure 5 shows the SEM images of the surface wear of the HPMC and HPMC-based composites. The appearance of surface wear is different for each additive. In the images of pure HPMC (Figure 5a), the Si surface of a small area has been damaged. This is because the HPMC thin film has been worn and its protection is lost, resulting in damage and wear of the base material during the late wear period [52]. In the IF-MoS_2_ additive coating images (Figure 5b), we can clearly see a layer of tribofilm. This film ensures a consistently low friction coefficient and good wear resistance capability. In the 2H-MoS_2_ additive coating images (Figure 5c), obvious particle debris and destruction of the Si surface in a small area can be observed. It is speculated that these particle debris contain a large amount of Si substrate wear debris. This may cause abrasive wear to the transfer film or hinder its stable formation and can also result in abrasive wear on the substrate. Similarly, Si debris and a fairly large area of Si surface damage can also be seen in the images of the MoO_3_ additive coating (Figure 5d), indicating that abrasive wear is also very likely to occur during friction.

**Figure 6:**
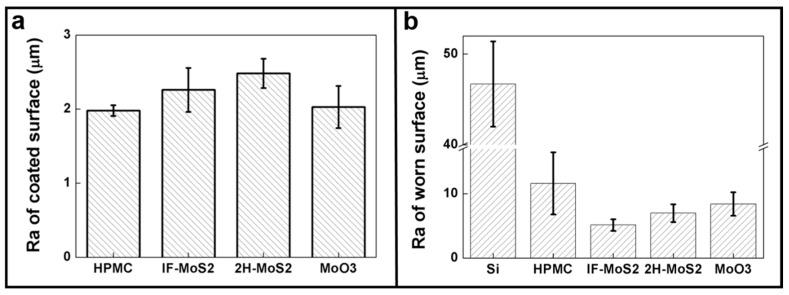
Surface roughness of (**a**) coated surface and (**b**) worn surface after tribotest.

The relationship between additive shape, size, and surface roughness significantly affects whether additives can deliver the best lubrication effect. For counter materials with a smooth surface, adding small-sized particle additives can achieve a good lubrication effect [26,28]. Figure 6a shows that the surface roughness of the thin film of all the composites is about 2 μm after the additive is added; thus, the effect of surface roughness on tribological behavior can be neglected. Moreover, since the surface of the counter chromium steel ball is smooth, the small-sized particles can provide effective lubrication [27]. Figure 6b shows the surface roughness of the specimens after wear. The surface roughness of the IF-MoS_2_ additive specimen is the best. One possible reason is that this additive has the smallest size, and the debris after wear (particles may be crushed or stripped, so the debris size may be smaller than that of the particles [55,56]) is mixed in the third body layer formed by wear debris, flowing as a third body [58,59]. As the friction process progresses, this third body is likely to enter the contact area to bear the load by separating the asperities of the friction surfaces to reduce the adsorption force of the friction interface [28], or by forming a transfer layer with excellent adhesive force to prevent direct contact between the asperities of the test pieces and the counter material. In either case, the base materials are protected [37,60]. Compared with IF-MoS_2_, the 2H-MoS_2_ additive forms particles and debris with larger sizes, which cannot easily enter the contact area and thus provide less protection for the base materials.

In addition to the effects of size, the shape and structure of particles also affect their lubrication mechanisms, greatly affecting the bulk tribological performance [51,61]. A comparison of IF-MoS_2_ and MoO_3_ particles with those of a similar size reveals the following: Since IF-MoS_2_ is spherical in shape while MoO_3_ has an irregular flaky shape, the wear debris formed by IF-MoS_2_ provides an additional rolling mechanism in the third body flow to provide more velocity accommodation modes, enabling this additive to exhibit better tribological properties. The sharp corners of MoO_3_ particles may easily cause abrasive wear to hinder the formation and development of the transfer film, thus delivering poor wear resistance [27,56,57].

**Figure 7:**
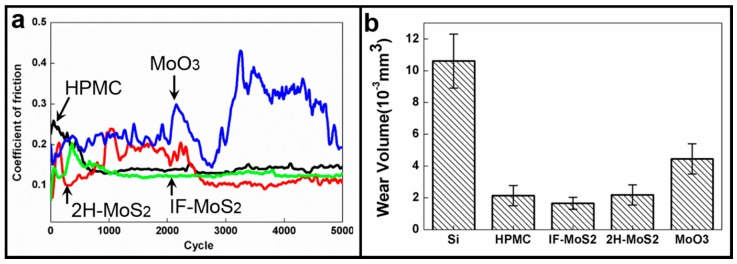
(**a**) COF results of pure HPMC and IF-MoS_2_ NP-, 2H-MoS_2_ NP-, and MoO_3_ NP-added HPMC; (**b**) wear volume of Si, pure HPMC, and IF-MoS_2_-, 2H-MoS_2_-, and MoO_3_-added HPMC.

The COFs have been compared between IF-MoS_2_ and 2H-MoS_2_ in Figure 7a, and the COF values can be as low as 0.1, which is higher than the expected value (0.03–0.05). The unexpected COFs shown by IF-MoS_2_ and 2H-MoS_2_ particles might be caused by the low contact stress and low temperature, thus, the MoS_2_ particles are probably still inert for chemical reaction to demonstrate their spectacular lubricity [62]. However, the COF shown by IF-MoS_2_ NPs are relatively more stable. In the HPMC system, it is easy to observe the tribological behaviors in three stages. In the first stage (referred to as the initial stage), the COF goes up quickly; no transfer layer is generated in the HPMC material; hence, the increase in COF is observed. In the second stage (referred to as the running-in period), the transfer layer is in an unstable state (it is increased and removed alternately). In the third stage (referred to as the stable period), the COF is stable and moderate; the stably generated transfer layer can provide favorable tribological properties effectively. The 2H-MoS_2_ has a clear running-in period. It is difficult for the large-sized additives to enter the wear zone during wear. Therefore, the generation and coverage of the transfer layer are not stable; hence, it causes an unstable rise in the COF.

As mentioned before, one possible reason is that their relatively small size enables the abrasive particles of IF-MoS_2_ to enter the contact area more easily. The mechanism of the lubrication provided by additives is also an important factor affecting the tribological properties of composites [56,57]. MoO_3_, with its lamellar structure [63,64], has been regarded as a potential solid lubricant. Its lubricating mechanism is mainly sliding [65,66]. However, IF-MoS_2_ has more friction reduction mechanisms, such as rolling, sliding, exfoliation, and fracture, leading to better lubricating performance under a wide range of stresses [56].

A HPMC thin film has demonstrated excellent lubricating properties and can effectively minimize COF and protect the substrate from wear [53]. The excellent lubricating properties of HPMC have been achieved using the transfer layer, which adhered to counter surfaces and can be gradually exhausted due to wear. When most of the HPMC is exhausted and the thin film is worn out, COF will suddenly increase and become unstable [43,53]. The lubricating properties of HPMC film can be improved by adding appropriate additives [47]. Figure 7b shows the wear volume of bare silicon, pure HPMC, and HPMC with three added additives. The HPMC coating can, to a significant extent, protect the substrate from abrasion and reduce the abrasion of Si-based substrates by more than 80%. Comparing the wear resistance among different additives, it was found that HPMC with IF-MoS_2_ NPs demonstrated the lowest wear. A MoS_2_ additive (both IF-MoS_2_ and 2H-MoS_2_ additives) can reduce wear and COF 50% more than MoO_3_. IF-MoS_2_ additive results in highly improved lubricating properties because its debris has can easily enter the contact area and produce thin and well-covered transfer films (as shown in Figure 5b), which develops self-lubricating properties in MoS_2_-MoS_2_ and therefore leads to low and stable COFs [51,67,68,69], protecting the substrate. In contrast, despite the fact that the debris particles formed by MoO_3_ additives are probably also sufficiently small to enter the contact area, they slightly increase substrate wear and provide a higher COF. Their higher hardness, irregular edges, and the corners of MoO_3_ NPs hinder the formation and development of a stable transfer film and damage the substrate surface, increasing wear volume [27,28]. Owing to its multiple friction mechanisms and the capacity to produce and develop a stable transfer layer, IF-MoS_2_ provides the lowest and most stable friction coefficient and wear volume, which are greatly correlated with the size, shape, and structure of the NPs. The experiment results show that proper additives can improve the tribology performance of HPMC thin films.

## 4. Conclusions

(1) High-density composite material MoS_2_/MoO_3_ particles were successfully produced via the simple sulfidation method.
(2) The thin film of lubricating material was produced with excellent homogeneity from the biological polymer material HPMC with NP additives.
(3) MoS_2_ particles were added to HPMC, and friction and wear were reduced by more than 50%. A more stable COF was also achieved when MoO_3_ was added.
(4) The study showed that IF-MoS_2_ NPs have multiple lubricating mechanisms, and superior and more stable lubricating properties compared with 2H-MoS_2_ MPs and MoO_3_ NPs.
(5) It is hypothesized that the IF-MoS_2_/HPMC thin film showed the best tribological performance in our experiment because its wear debris can easily enter the contact area to form a tribofilm/transfer film with good coverage; IF-MoS_2_ NPs have multiple friction mechanisms, which are unique to this additive’s size, shape, and structure.

## Figures and Tables

**Table 1:** Specifications of hydroxypropyl methylcellulose (HPMC).

Molecular Weight Mn	Methyl (CH_3_) Substitution (%)	Hydroxypropyl (CH_2_CHOHCH_3_) Substitution (%)	Viscosity (2 wt % Aqueous Solutions at 20 °C)
35,600	28.8	9	5.94 mPa·s

## Abbreviations

The following abbreviations are used in this manuscript:

NCKU	National Cheng Kung University
HPMC	hydroxypropyl methylcellulose
NP	nanoparticle
IF	inorganic fullerene
2H	hexagonal
COF	coefficient of friction
SEM	scanning electron microscope
EDS	energy dispersive spectroscopy
